# How good is the clinical diagnosis in schizophrenia? Reliability and validity

**DOI:** 10.1192/j.eurpsy.2024.60

**Published:** 2024-08-27

**Authors:** P. Falkai

**Affiliations:** Psychiatry, University of Munich, Munich, Germany

## Abstract

**Abstract:**

Several changes to the classification of mental disorders have been made during the past half century to increase the reliability, clinical use and validity of the diagnostic classification. Despite the high expansion of knowledge about mental disorders, understanding of their components and processes still requires fine-tuning. This symposium identifies key issues on different classification systems with different purposes relevant to understanding and classifying mental disorders. We discuss how key issues such as ICD-11, RDoC or Biomarkers correspond or diverge because of their different purposes, and constituencies. Although these approaches have varying degrees of overlap and distinguishing features, they share the goal of reducing the burden of suffering due to mental disorder.

**Disclosure of Interest:**

None Declared

